# Introduction to the *Toxins* Special Issue on “Novel Issues in Uremic Toxicity”

**DOI:** 10.3390/toxins10100388

**Published:** 2018-09-25

**Authors:** Raymond Vanholder

**Affiliations:** Nephrology Section, 0K12, University Hospital, De Pintelaan, 185, B9000 Gent, Belgium; raymond.vanholder@ugent.be; Tel.: +32-475-512-751; Fax: +32-933-24599

## 1. Introduction

When kidney functions fail (chronic kidney disease—CKD), this results in a diminished urinary excretion and thus retention of waste products of metabolism and a gradual decline of almost every function of the body. This process is attributed to an endogenous intoxication by these accumulated metabolites. This emanates in a clinical picture that is called “uremic syndrome,” a term interchangeably used with “uremia” and referring to the most abundant and also first recognized retention product, urea. In as far as elements of the clinical picture are caused by the biological or biochemical action of these retained metabolites, the latter are called “uremic toxins,” and their consequence is “uremic toxicity.”

The clinical picture emanating from this uremic syndrome as well as the mechanisms leading to it are complex and multifactorial. Major patho-physiologic mechanisms at play are inflammation, protein energy wasting, disturbed glucose handling, dysfunction of vascular cells, thrombogenesis, and fibrosis, but the list of baseline mechanisms is far more extensive [1]. The leading organ dysfunction and a major cause of death in CKD patients is cardiovascular disease [2], but in this respect, the picture is more exhaustive, comprising susceptibility to infection, gastro-intestinal dysfunction, inadequate handling of metabolites, and neural conduction disturbances [1]. A newly detected and likely important contributor is the role played in uremic toxin generation by the intestine and the intestinal microbiome [3,4] that affects and is affected by the uremic status.

The knowledge of uremic toxicity (as well of the responsible retention products, of their retention pattern as of their toxic effects) has been growing exponentially over the last decades, in part stimulated by the publication of encyclopedic uremic toxin lists [5,6], the novel possibilities raised by the refinement of comprehensive “omic” approaches [7,8], and the creation of collaborative work groups focusing on uremic toxicity such as the European Uremic Toxin Work Group (EUTox) [9]. In the slipstream of our increasing knowledge of the components exerting uremic toxicity, and as a consequence of the increasing possibilities of biochemistry and molecular biology, also our understanding of the mechanisms at the origin of uremic toxicity has been growing. 

In this context, it was decided in 2015–2016 that the journal *Toxins* would publish a special issue on uremic toxicity that would be devoted to the novel uremic toxins, either newly detected molecules, or well-known solutes with newly detected biological effects. Focus could be as well on identification, biologic effects, generation as on removal or other approaches to decrease concentration. It is the intention of this editorial to summarize the content of the 15 publications contained in this special issue. To reach this aim, toxins will be discussed according to the currently used classification system, i.e., small water-soluble compounds, protein bound compounds and middle molecules (Table 1).

The special issue contains reviews, essentially about relatively recently identified and important uremic toxins, or about toxins that were known since long but received a renewed interest recently because of a shift in paradigm about their toxicity. In addition, this special issue also contains a number of peer-reviewed original publications, one letter to the editor, and one comprehensive review covering the broad overall picture of uremic toxicity.

## 2. Small Water-Soluble Compounds

### 2.1. Trimethylamine-N-Oxide (TMAO)

Trimethylamine-*N*-oxide (TMAO) is known as a chemical protector against the protein destabilizing effect of urea but has only recently been recognized as well as an agent with major toxic potential. In a metabolomic analysis, TMAO was identified as a strong predictor of cardio-vascular risk and subsequent experimental studies demonstrated its pro-atherogenic impact [10]. In several clinical studies in the general population, its concentration was associated to cardio-vascular morbidity and mortality [10]. Later on, it was also demonstrated that this compound was a uremic retention product and that its concentration was linked to negative hard outcomes in the CKD population. Interestingly, like many other uremic toxins, also TMAO appears to be the product of metabolic transformation of food elements by the intestinal microbiome, and in the conceptual development of strategies to decrease their concentration, impacting the intestinal status is an option that is currently considered [10]. Yet, it presently remains debated whether this compound is a culprit or just a marker, in view of a number of paradoxical observations, such as its high concentrations in fish, while dietary fish intake obviously is not a factor contributing to enhanced cardiovascular risk. Thus, further study is needed to establish all molecular pathways that are impacted by TMAO [10].

### 2.2. Lanthionine

Lantionine is a sulfur-containing non-proteinogenic amino acid resulting from the condensation of two cysteine molecules, whereas related homolanthionine results from the condensation of two homocysteine molecules [11]. In uremia, the concentrations of both homolanthionine and especially lanthionine are increased manifold. Sulfur metabolites have been recognized already since a few decades to play a potential role in uremic cardio-vascular disease, whereby up till now most attention has been paid to homocysteine, which is well recognized as a potential cardio-vascular risk factor in the general population. However, lanthionine may also contribute to uremic cardio-vascular risk by increasing homocysteine concentration and inhibiting hydrogen sulfide production [11]. The latter is a volatile compound to which a protective role on the cardio-vascular system has been attributed, as it is a vasorelaxant with antihypertensive properties. Its deficiency, on the other hand, has been linked to atherogenesis [11]. The authors of the review included in this special issue therefore suggest that lanthionine should be considered as a novel uremic toxin [11].

### 2.3. N-Methyl-2-pyridone-5-carboxamide (2PY)

*N*-methyl-2-pyridone-5-carboxamide (2PY) is the subject of another review [12]. 2PY is a pyridine derivative of nicotinamide, in its turn a water-soluble amine product of nicotinic acid. The molecule was classified as a uremic retention product many years ago [5] but gained renewed interest with the exploration of the potential of nicotinamide for phosphate control in end stage kidney disease (ESKD), which also induces a manifold increase in 2PY concentration [12]. The review describes how the compound has a toxic potential, mainly by inhibiting poly(ADP-ribose) polymerase-1 (PARP-1), possibly leading to genomic instability or tumorigenesis, but PARP-1 inhibition also has the potential capacity to prevent or attenuate some acute processes such as stroke, myocardial infarction, and septic shock [12]. 2PY has also been related to thrombocytopenia and leukopenia. In several studies in hemodialysis populations, a number of patients treated with nicotinamide developed thrombocytopenia, although the role of 2PY in this effect is not well established, underscoring the current need for further assessment of its possible biological and clinical impact [12].

### 2.4. Uric Acid

In a combined clinical and experimental study, the relationship between uric acid and mortality on one hand (clinical arm) and uric acid and inflammation (experimental arm) was assessed [13]. In the clinical part of the study considering a large hemodialyzed population, an inverse association between uric acid and mortality was observed, with the highest mortality in the quartile with the lowest uric acid. In the experimental arm, in vitro studies indicated that the pro-inflammatory effect (oxidative stress) and inhibition of nitric oxide synthesis induced by indoxyl sulfate were attenuated by uric acid [13]. The clinical data may seem in contradiction with other studies, pointing to a positive correlation between uric acid concentration and cardio-vascular morbidity and mortality [24], but the hemodialysis population in which the study was undertaken may differ in its characteristics from patients with CKD, in whom most of this type of outcome studies has been undertaken. In addition, the studied population was Taiwanese, which may make them different from other populations both genetically and metabolically, and residual confounding cannot be excluded. On the other hand, the attenuation of the toxic effect of indoxyl sulfate may be explained by the fact that uric acid at certain concentrations may act as an antioxidant. 

## 3. Protein Bound Compounds

### 3.1. Advanced Oxidation Protein Products (AOPPs)

Similar to advanced glycation end products (AGEs), advanced oxidation protein products (AOPPs) are also posttranslational protein modifications of both complete proteins and protein degradation products induced by oxidative stress. Both AOPPs prepared in vitro and AOPPs extracted from plasma of hemodialysis patients have been linked to pro-inflammatory mechanisms. In the original experimental study on AOPPs included in this special issue, it was shown that plasma AOPPs affected the innate immune cell phenotype and function by altering the thiol-redox equilibrium, and in this way may be involved in the migration, accumulation and even proliferation of immune cells such as dendritic cells or macrophages into atherosclerotic plaque, contributing to progression of vascular disease in patients with advanced kidney disease [14].

### 3.2. Indoxyl Sulfate

Not surprisingly, and conforming with the broad attention indoxyl sulfate received in the literature of the last two decades, a large number of articles in this special issue are devoted to this solute.

First of all, the issue includes a comprehensive review on a large array of aspects related to this prototypic protein bound uremic toxin, a small molecule with more than 90% protein binding and originating from the intestinal metabolism of tryptophan [15]. The kidneys provide a high clearance of this solute via tubular secretion, while removal by hemodialysis compared to this is low, resulting in an exponential accumulation in dialysis patients. A host of experimental studies have inculpated indoxyl sulfate with a role in progression of CKD and vascular disease, but also in low bone turnover disease and disturbances of the central nervous system [15]. Toxicity of indoxyl sulfate in humans has not directly been proven, as most clinical data linking its concentration to outcomes are observational [15]. In view of the dismal removal by dialysis, this has resulted in a number of alternative strategies such as intestinal adsorption by AST-120 (Kremezin^®^, Kureha Chemical, Tokyo, Japan). When the latter compound was subjected to a large RCT, no impact on progression of CKD could be demonstrated [25], and controlled studies of the impact of lowering indoxyl sulfate concentration on survival or cardiovascular events are as yet lacking. As a consequence, it remains difficult to make definite conclusions about the toxicity of this compound [15].

A letter to the editor that is also included in this special issue, offers an addendum to the above review, pointing out a number of studies demonstrating a link between indoxyl sulfate concentration and biochemical and histomorphometric parameters of low bone turnover in CKD patients [16].

A second review focuses specifically on the role of indoxyl sulfate in peripheral artery disease and dialysis graft thrombosis [17], as well as on newly detected mechanisms that could be at play in these processes, such as progenitor cell-related neovascularization and tissue factor-related hypercoagulability [17]. The authors raise the possibility that strategies targeting at a decrease of indoxyl sulfate concentration may have the potential to improve outcomes related to peripheral vascular disease and dialysis vascular access thrombosis [17].

An original publication follows the same line of thought and assesses in animal models the role of indoxyl sulfate in accelerating thrombotic response to vascular injury [18]. The study assessed the impact of an acute administration of indoxyl sulfate on thrombus formation induced by vascular lesions caused by an electric current and laser damage to the endothelium [18]. The study showed a dose dependent increase in risk of thrombus formation. The authors also found a decrease in clotting time and an increase in maximal clot firmness in the presence of indoxyl sulfate [18]. These data point to the occurrence of a pro-thrombotic status in the presence of indoxyl sulfate.

In a study essentially focusing on the impact of uric acid to counter the effect of indoxyl sulfate (see above), another article in this special issue shows that indoxyl sulfate increases oxidative stress and inhibits nitric oxide synthesis in human aortic endothelial cells [13]. These findings, at least with regard to the effect of indoxyl sulfate alone, are merely a confirmation of previously reported data [26]. The originality of this publication rather lies in the description of the impact of uric acid on this effect.

Another study also assesses the proinflammatory impact of indoxyl sulfate, and demonstrates in vitro macrophage activation through multiple pathways including activation of the aryl hydrocarbon receptor [19]. In this way, the study suggests that the aryl hydrocarbon receptor is not only involved in hypercoagulability but also in inflammation. Unfortunately, the study utilizes indoxyl sulfate concentrations that are considerably higher than those observed in CKD. The authors argue that indoxyl sulfate concentration may be that high in atherosclerotic plaque, but this statement is hypothetical and needs to be proven. 

### 3.3. P-Cresylsulfate

One of the reviews in this special issue is devoted to p-cresyl sulfate [20]. This molecule made its appearance in the list of uremic toxins only recently, when it appeared that the presumed presence in samples of CKD patients of p-cresol, up till then considered the main cresol present in uremia, appeared to be an artifact. In reality, its conjugates (p-cresyl sulfate as well as other cresyl derivatives) are the major cresols retained in uremia [27,28,29]. Increased levels of p-cresyl sulfate have been associated with worsening outcomes in CKD patients [20]. The article also pays ample attention to the origin of this compound, which is generated by the intestinal microbiota by metabolism of aromatic amino acids, such as tyrosine and phenylalanine, leading to a whole spectrum of phenolic compounds, of which p-cresyl sulfate is the compound with the highest concentration [20]. As p-cresyl sulfate remains difficult to remove by extracorporeal treatment, influencing gut microbiota might be a possible option in future to decrease the concentration and the toxicity of this compound, even at early stages of CKD [20].

## 4. Middle Molecules

### 4.1. Fibroblast Growth Factor-23 (FGF-23)

One of the reviews in this issue is devoted to fibroblast growth factor-23 (FGF-23) [21]. FGF-23 has been introduced in the spectrum of uremic retention solutes only during the last 1.5 decades and is best known as a regulator of calcium-phosphate homeostasis. FGF-23 is one of the first elements in this chain to increase in concentration, initially with as main role to keep serum phosphorus stable in spite of a decrease in GFR by also decreasing renal reabsorption of phosphate and 1,25(OH)_2_ vitamin D generation in the kidneys [21]. This effect probably comes at the cost of an increased negative biological impact to the body. FGF-23 concentration has been linked to negative hard outcomes such as progression of CKD, overall mortality, and cardio-vascular morbidity and mortality in a host of studies [21]. Yet, those associations offered no direct proof of causation, and until recently, it has been debated whether this compound was just a simple marker without biological impact or a real uremic toxin. However, recent experimental work indicated a biological impact, especially in generating cardiac hypertrophy and inflammation. Although there are several options to decrease FGF-23 concentration in CKD (dietary phosphate restriction, intestinal phosphate binders, calcimimetics, and nicotinamide), the most efficient method to normalize FGF-23 remains a successful kidney transplantation [21]. 

### 4.2. Modified Lipids and Lipoproteins

One of the publications included in this special issue focuses on a group of molecules that is present in increased concentration in uremia but that is rarely mentioned in reviews on uremic toxins: the modified lipids and lipoproteins [22]. Like with advanced glycation end products (AGEs) and advanced oxidation protein products (AOPP) (see elsewhere in this editorial), these are posttranslational modifications as a consequence of the enhanced oxidative stress of uremia. These modified compounds exert toxic effects on cells and tissues, e.g., by inducing pro-apoptotic and pro-inflammatory effects [22]. Some of these modified lipids, like the F2-isoprostanes, are directly related to CKD progression [22]. Modifications of high density lipoprotein (HDL) also play an important role in altering the physiologic effect of these molecules, resulting in accelerated arteriosclerosis and a blunted effect of statins in CKD, especially in ESKD [22]. 

### 4.3. Cytokines

Finally, one last review considers the patho-physiologic role of cytokines in uremia and the information gaps that still exist regarding their clinical impact in CKD patients [23]. The authors also discuss the potential of cytokines to contribute to the manifestations and complications of CKD and the relationship of those concentrations with negative outcomes. The impact of therapeutic interventions to reduce the concentration or impact of these cytokines is also discussed, stressing the relative scarcity at present of this type of data [23]. The biochemical impact of several cytokines, such as interleukin-1β (IL-1β), interleukin-6 (IL-6), interleukin-18 (IL-18), and tumor necrosis factor-α (TNF-α), is stressed [23]. At the same time, some other solutes like leptin, resistin, interleukin-10 (IL-10), and adiponectin are discarded as not being real uremic toxins [23]. 

## 5. Comprehensive Review on Uremic Toxins at Large

The closing publication in this special issue is a comprehensive narrative review of the most important uremic toxins and their toxic effects, especially those not directly discussed in the other publications of the issue [24]. A large array of solutes in each major group (small water soluble, protein bound and middle molecules) was considered regarding their effect on 11 biological and organ systems. Evidence appeared to be especially focused on cardio-vascular damage, inflammation and fibrosis but these were probably also the systems that had been most often evaluated [24]. A scoring system was developed to classify the reviewed compounds according to the experimental evidence of their toxicity (number of systems affected) and overall clinical and experimental evidence. The highest globally scoring solutes are specified in Table 2. More experimental data have been provided in the literature for protein bound molecules, but in spite of these robust experimental data, clinical evidence is missing for almost half them, pointing to a deficiency in clinical studies of those protein bound solutes [24]. The emerging picture points to uremia as a complex condition, where a host of factors contribute to a multisystem complication profile. Therapeutic approaches should take into consideration this complexity and should probably not focus on one single solute or group of solutes [24]. 

## 6. Conclusions

We are convinced that this special issue of *Toxins* devoted to uremic toxicity will offer a kaleidoscopic image to the readership of the journal about the complex and intriguing clinical picture that is the consequence of uremic toxins. Within the scope of the journal, uremic toxicity occupies a peculiar place. In contrast to most toxins discussed in this journal, uremic toxicity is not exogenous but largely endogenous, i.e., caused by substances that typically are generated in the body, whereby the increased concentration to a large extent is the consequence of the decreased removal by the failing kidneys.

During the development of this special issue, the editors of the journal decided to create a specific section devoted to uremic toxins [30], encouraging the submission of articles on the identification, characterization, biological effect, generation, complications, removal, kinetics, genomics, proteomics, and metabolomics related to uremic toxins, as well as on the impact of the intestinal microbiome on their activity and the many other aspects that potentially are related to this topic, and this in the contexts of chronic kidney disease, dialysis treatment, transplantation, and acute kidney injury. In the same line, the initiative was taken to launch two special issues: one on uremia and cardiovascular disease with Z Massy as a guest editor [31] and one on the intestine and uremia with G Glorieux as guest editor [32]. We hope that the current comprehensive issue that now comes to a close, will be a stimulus to boost submissions to the uremic toxin section of *Toxins* and the two special issues mentioned above. 

## Figures and Tables

**Table 1:** Articles contained in the special issue on “novel uremic toxins.”

Author Name	Title	Toxin(s) Discussed	Reference	Reference in This Editorial	Country of Origin
Velasquez	Trimethylamine-*N*-oxide: the good, the bad and the unknown	TMA0	*8*, 326, 2016	[10]	USA
Perna	The sulfur metabolite lanthionine: evidence for a role as a novel uremic toxin	Lanthionine	*9*, 26, 2017	[11]	Italy
Lenglet	*N*-methyl-2-pyridone-5-carboxamide (2PY)—major metabolite of nicotinamide: an update on an old uremic toxin	2PY	*8*, 339, 2016	[12]	France
Hsu	High uric acid ameliorates indoxyl sulfate-induced endothelial dysfunction and is associated with lower mortality among hemodialysis patients	Uric acid IS	*9*, 20, 2017	[13]	Taiwan
Garibaldi	Advanced oxidation protein products-modified albumin induces differentiation of RW264.7 macrophages into dendritic-like cells which is modulated by cell surface thiols	AOPPs	*9*, 27, 2017	[14]	Italy
Leong	Indoxyl sylfate—review of toxicity and therapeutic strategies	IS	*8*, 358, 2016	[15]	USA
Barreto *	Comment on indoxyl sulfate—review of toxicity and therapeutic strategies. Toxins 2016, *8*, 358	IS	*9*, 142, 2017	[16]	Brazil
Wu	Impact of indoxyl sulfate on progenitor cell-related neovascularization of peripheral artery disease and post-angioplasty thrombosis of dialysis vascular access	IS	*9*, 25, 2017	[17]	Taiwan
Karbowska	The uremic toxin indoxyl sulfate accelerates thrombotic response after vascular injury in animal models	IS	*9*, 229, 2017	[18]	Poland
Wakamatsu	Indoxyl sulfate promotes macrophage IL-1 production by activating aryl hydrocarbon receptor/NF-κ/MAPK cascades, but the NLRP3 inflammasome was not activated	IS	*10*, 124, 2018	[19]	Japan
Gryp	P-cresyl sulfate	PCS	*9*, 52, 2017	[20]	Belgium
Kuczera	Fibroblast growth factor-23—A potential uremic toxin	FGF-23	*8*, 369, 2016	[21]	Poland
Florens	Modified lipids and lipoproteins in chronic kidney disease: a new class of uremic toxins	ML&L	*8*, 376, 2016	[22]	France
Castillo-Rodriguez	Inflammatory cytokines as uremic toxins: “Ni son todos los que estan, ni estan todos los que son”	Cytokines	*9*, 114, 2017	[23]	Spain
Vanholder	Biochemical and clinical impact of organic uremic retention solutes: a comprehensive update	All	*10*, 33, 2018	[24]	Belgium

TMAO: Trimethylamine-*N*-oxide; 2PY: *N*-methyl-2-pyridone-5-carboxamide; IS: indoxyl sulfate; AOPPs: advanced oxidation protein products; PCS: p-cresyl sulfate; FGF-23: fibroblast growth factor-23; ML&L: modified lipids and lipoproteins.*: letter to the editor.

**Table 2:** Solutes with the highest scores for overall evidence level *.

− **Small water soluble compounds**
o Asymmetric dimethylarginine (ADMA)
o Trimethylamine-*N*-oxide (TMAO)
o Uric acid
− **Protein bound compounds**
o Advanced glycation end products (AGEs)
o P-cresyl sulfate
o Indoxyl sulfate
o Indole acetic acid
o The kynurenines
o Phenyl acetic acid
− **Middle molecules**
o B_2_-microglobulin
o Ghrelin
o Parathyroid hormone

*: list extracted from Vanholder et al. [24], Toxins, *10*, 33, **2018**.

## References

[1] Vanholder R., Fouque D., Glorieux G., Heine G.H., Kanbay M., Mallamaci F., Massy Z.A., Ortiz A., Rossignol P., Wiecek A. (2016). Clinical management of the uraemic syndrome in chronic kidney disease. Lancet Diabetes Endocrinol..

[2] Vanholder R., Massy Z., Argiles A., Spasovski G., Verbeke F., Lameire N., European Uremic Toxin Work Group (2005). Chronic kidney disease as cause of cardiovascular morbidity and mortality. Nephrol. Dial. Transplant..

[3] Vanholder R., Glorieux G. (2015). The intestine and the kidneys: A bad marriage can be hazardous. Clin. Kidney J..

[4] Schepers E., Glorieux G., Vanholder R. (2010). The gut: The forgotten organ in uremia?. Blood Purif..

[5] Vanholder R., De Smet R., Glorieux G., Argilés A., Baurmeister U., Brunet P., Clark W., Cohen G., De Deyn P.P., Deppisch R. (2003). Review on uremic toxins: Classification, concentration, and interindividual variability. Kidney Int..

[6] Duranton F., Cohen G., De Smet R., Rodriguez M., Jankowski J., Vanholder R., Argiles A., European Uremic Toxin Work Group (2012). Normal and pathologic concentrations of uremic toxins. J. Am. Soc. Nephrol..

[7] Rhee E.P., Souza A., Farrell L., Pollak M.R., Lewis G.D., Steele D.J., Thadhani R., Clish C.B., Greka A., Gerszten R.E. (2010). Metabolite profiling identifies markers of uremia. J. Am. Soc. Nephrol..

[8] Mischak H., Delles C., Vlahou A., Vanholder R. (2015). Proteomic biomarkers in kidney disease: Issues in development and implementation. Nat. Rev. Nephrol..

[9] The European Uremic Toxin Work Group (EUTox). http://www.uremic-toxins.org/.

[10] Velasquez M.T., Ramezani A., Manal A., Raj D.S. (2016). Trimethylamine N-Oxide: The Good, the Bad and the Unknown. Toxins.

[11] Perna A.F., Zacchia M., Trepiccione F., Ingrosso D. (2017). The Sulfur Metabolite Lanthionine: Evidence for a Role as a Novel Uremic Toxin. Toxins.

[12] Lenglet A., Liabeuf S., Bodeau S., Louvet L., Mary A., Boullier A., Lemaire-Hurtel A.S., Jonet A., Sonnet P., Kamel S. (2016). N-methyl-2-pyridone-5-carboxamide (2PY)-Major Metabolite of Nicotinamide: An Update on an Old Uremic Toxin. Toxins.

[13] Hsu W.L., Li S.Y., Liu J.S., Huang P.-H., Lin S.-J., Hsu C.-C., Lin Y.-P., Tarng D.-C. (2017). High Uric Acid Ameliorates Indoxyl Sulfate-Induced Endothelial Dysfunction and Is Associated with Lower Mortality among Hemodialysis Patients. Toxins.

[14] Garibaldi S., Barisione C., Marengo B., Ameri P., Brunelli C., Balbi M., Ghigliotti G. (2017). Advanced Oxidation Protein Products-Modified Albumin Induces Differentiation of RAW264.7 Macrophages into Dendritic-Like Cells Which Is Modulated by Cell Surface Thiols. Toxins.

[15] Leong S.C., Sirich T.L. (2016). Indoxyl Sulfate-Review of Toxicity and Therapeutic Strategies. Toxins.

[16] Barreto F.C., Barreto D.V., Stinghen A.E.M., Massy Z.A. (2017). Comment on Indoxyl Sulfate-Review of Toxicity and Therapeutic Strategies. Toxins.

[17] Wu C.C., Hung S.C., Kuo K.L., Tarng D.C. (2017). Impact of Indoxyl Sulfate on Progenitor Cell-Related Neovascularization of Peripheral Arterial Disease and Post-Angioplasty Thrombosis of Dialysis Vascular Access. Toxins.

[18] Karbowska M., Kaminski T.W., Marcinczyk N., Misztal T., Rusak T., Smyk L., Pawlak D. (2017). The Uremic Toxin Indoxyl Sulfate Accelerates Thrombotic Response after Vascular Injury in Animal Models. Toxins.

[19] Wakamatsu T., Yamamoto S., Ito T., Sato Y., Matsuo K., Takahashi Y., Kaneko Y., Goto S., Kazama J.J., Gejyo F., Narita I. (2018). Indoxyl Sulfate Promotes Macrophage IL-1β Production by Activating Aryl Hydrocarbon Receptor/NF-κ/MAPK Cascades, but the NLRP3 inflammasome was Not Activated. Toxins.

[20] Gryp T., Vanholder R., Vaneechoutte M., Glorieux G. (2017). p-Cresyl Sulfate. Toxins.

[21] Kuczera P., Adamczak M., Wiecek A. (2016). Fibroblast Growth Factor-23-A Potential Uremic Toxin. Toxins.

[22] Florens N., Calzada C., Lyasko E., Juillard L., Soulage C.O. (2016). Modified Lipids and Lipoproteins in Chronic Kidney Disease: A New Class of Uremic Toxins. Toxins.

[23] Castillo-Rodriguez E., Pizarro-Sanchez S., Sanz A.B., Ramos A.M., Sanchez-Niño M.D., Martin-Cleary C., Fernandez-Fernandez B., Ortiz A. (2017). Inflammatory Cytokines as Uremic Toxins: “Ni Son Todos Los Que Estan, Ni Estan Todos Los Que Son”. Toxins.

[24] Vanholder R., Pletinck A., Schepers E., Glorieux G. (2018). Biochemical and Clinical Impact of Organic Uremic Retention Solutes: A Comprehensive Update. Toxins.

[25] Schulman G., Berl T., Beck G.J., Remuzzi G., Ritz E., Arita K., Kato A., Shimizu M. (2015). Randomized Placebo-Controlled EPPIC Trials of AST-120 in CKD. J. Am. Soc. Nephrol..

[26] Vanholder R., Schepers E., Pletinck A., Nagler E.V., Glorieux G. (2014). The uremic toxicity of indoxyl sulfate and p-cresyl sulfate: A systematic review. J. Am. Soc. Nephrol..

[27] Martinez A.W., Recht N.S., Hostetter T.H., Meyer T.W. (2005). Removal of P-cresol sulfate by hemodialysis. J. Am. Soc. Nephrol..

[28] De Loor H., Bammens B., Evenepoel P., De Preter V., Verbeke K. (2005). Gas chromatographic-mass spectrometric analysis for measurement of p-cresol and its conjugated metabolites in uremic and normal serum. Clin. Chem..

[29] Vanholder R., Bammens B., de Loor H., Glorieux G., Meijers B., Schepers E., Massy Z., Evenepoel P. (2011). Warning: The unfortunate end of p-cresol as a uraemic toxin. Nephrol. Dial. Transplant..

[30] A new section “Uremic Toxins” has been established in Toxins. http://www.mdpi.com/journal/toxins/announcements.

[31] Special issue “Uremia and Cardiovascular Disease” in Toxins. http://www.mdpi.com/journal/toxins/special_issues/uremia_disease.

[32] Special issue “The Intestine and Uremia” in Toxins. http://www.mdpi.com/journal/toxins/special_issues/intestine-uremia.

